# Processing faecal samples: a step forward for standards in microbial community analysis

**DOI:** 10.1186/1471-2180-14-112

**Published:** 2014-05-01

**Authors:** Alba Santiago, Suchita Panda, Griet Mengels, Xavier Martinez, Fernando Azpiroz, Joel Dore, Francisco Guarner, Chaysavanh Manichanh

**Affiliations:** 1 Vall d’Hebron Research Institute, Digestive System Research Unit, Passeig de la Vall d’Hebron 119-129, Barcelona 08035, Spain; 2 Instituto de Salud Carlos III, Centro de Investigación Biomédica en Red en el Área temática de Enfermedades Hepáticas y Digestivas, CIBERehd, Madrid, Spain; 3 INRA, Institut National de la Recherche Agronomique, Jouy en Josas 78350, France

**Keywords:** 16S ribosomal RNA, Faecal sample collection, Needs for standardisation, Diarrhoea, Bead-beating

## Abstract

**Background:**

The microbial community analysis of stools requires optimised and standardised protocols for their collection, homogenisation, microbial disruption and nucleic acid extraction. Here we examined whether different layers of the stool are equally representative of the microbiome. We also studied the effect of stool water content, which typically increases in diarrhoeic samples, and of a microbial disruption method on DNA integrity and, therefore, on providing an unbiased microbial composition analysis.

**Results:**

We collected faecal samples from healthy subjects and performed microbial composition analysis by pyrosequencing the V4 region of the 16S rRNA gene. To examine the effect of stool structure, we compared the inner and outer layers of the samples (N = 8). Both layers presented minor differences in microbial composition and abundance at the species level. These differences did not significantly bias the microbial community specific to an individual. To evaluate the effect of stool water content and bead-beating, we used various volumes of a water-based salt solution and beads of distinct weights before nucleic acid extraction (N = 4). The different proportions of water did not affect the UniFrac-based clustering of samples from the same subject However, the use or omission of a bead-beating step produced different proportions of Gram-positive and Gram-negative bacteria and significant changes in the UniFrac-based clustering of the samples.

**Conclusion:**

The degree of hydration and homogenisation of faecal samples do not significantly alter their microbial community composition. However, the use of bead-beating is critical for the proper detection of Gram-positive bacteria such as *Blautia* and *Bifidobacterium*.

## Background

In the 1680s, Anton van Leeuwenhoek used homemade microscopes to provide the first description of faecal bacteria. Faecal specimens contain one of the densest microbial communities known, they have been shown to contain similar microbial community than the colon [1] and do not require an invasive collection protocol. Therefore they continue to be the samples most widely used for studying the intestinal microbiome, a collection of microbial genomes. In the last ten years, the greatest insights into the human intestinal microbiome have come about as a result of the application of metagenomics approaches to faecal samples, as attested by more than 1294 scientific publications found under the terms “human faecal microbiome” and “human fecal microbiome ” in PubMed.

Metagenomics approaches in biomedicine seek to provide a comprehensive picture of the diversity and abundance of dominant and subdominant microbial species in health [2,3] and in diseased states such as inflammatory bowel disorders (IBDs), irritable bowel syndrome (IBS) and other functional bowel disorders (FBD) [4-7]. During the course of these diseases, stool consistency is altered, varying from very hard (in constipation) to entirely liquid (in diarrhoea), as determined by the Bristol stool scale [8].

Diarrhoea is defined as an abnormally frequent discharge of semi-solid or fluid faecal matter from the bowel. As such, it usually implies a large percentage of water. A normal stool sample is considered to have a water content of about 75%, while that of a diarrhoeic stool is > 85% [9]. The freezing of specimens containing water causes the formation of ice crystals, which damage the microbial cell wall. Consequently, there is an increased release of cellular components such as DNase and RNase, which in turn may degrade nucleic acids at the beginning of the DNA extraction procedure. In intestinal disorders, such as IBD, IBS, and infectious diseases, the sampling of diarrhoeic stools is common [10,11]. However, how the water content of these samples affects the integrity of microbial DNA, and therefore the analysis of microbial composition, is unclear.

Steps such as stool homogenisation during collection or mechanical cell wall breaking during DNA extraction may affect the analysis of the microbial community. To date, no study on stool homogenisation or mechanical cell wall breaking using high-throughput sequencing technique has been reported. An appropriate collection protocol, together with a better understanding of the characteristics of a stool, is critical for downstream microbial community analysis.

Here we tested various factors that may affect microbial community analysis during stool sample collection and DNA extraction steps using gel electrophoresis and pyrosequencing of the 16S rRNA gene. In this regard, we examined the effect of homogenising the stool before freezing, the addition of a physiological solution to the stools to simulate a diarrhoeic condition before freezing, and the use of beads to breakdown the microbial cell wall during DNA extraction.

## Results and discussion

### Experimental design

Faecal samples were collected from healthy volunteers (n = 8) who had not taken antibiotics during the previous three months. Fresh samples were aliquoted as described below.

To test whether different layers of a stool sample unequally represent the microbiome, we compared the microbial composition of each faecal sample in three conditions: fully homogenised during sample collection, non-homogenised outer layers, and non-homogenised inner layers. For this comparison, two aliquots from each volunteer (#1 to #8, named L1 to L8) and for each condition were used. Thus, a total of 48 samples were prepared for microbial composition analysis.

To evaluate the effect of stool water content and the bead-beating technique on the integrity of microbial DNA and, therefore, on microbial composition analysis, fresh stool samples were homogenised with an increasing proportion of phosphate-buffered saline (PBS), as indicated in Table 1. Assuming that a normal stool contains 75% (range 56.6%–84.9%) of water, the dilutions tested corresponded to 75%, 80%, 87.5%, 93.8%, 97.5% and 99.5% of water content, respectively, which reflect the range of typical diarrhoeic samples [9,12]. Similar amounts of each diluted sample were then disrupted with and without a bead-beating step. This procedure was carried out for four of the eight volunteers cited above (#1, #3, #5 and #8, named DL1, DL3, DL5 and DL8). Thus, a total of 46 samples were collected for microbiome analysis.

**Table 1:** Addition of PBS to obtain stools with a range of water content

**ID**	**Weight (mg)**	**Presence of beads**	**PBS (μl)**	**Water content**
L#	250	yes	-	75.0%
DL#.00	250	yes	0	75.0%
DL#.25	187.5	yes	62.5	80.0%
DL#.50	125	yes	125.0	87.5%
DL#.75	62.5	yes	187.5	93.8%
DL#.90	25	yes	225.0	97.5%
DL#.98	5	yes	245.0	99.5%
DL#B.00	250	yes	-	75.0%
DL#B.25	187.5	yes	-	75.0%
DL#B.50	125	yes	-	75.0%
DL#B.75	62.5	yes	-	75.0%
DL#B.90	25	yes	-	75.0%
DL#B.98	5	yes	-	75.0%
DL#P.50	125	-	125.0	87.5%
DL#P.75	62.5	-	187.5	93.8%
DL#P.90	25	-	225.0	97.5%
DL#P.98	5	-	245.0	99.5%
DL#C.50	125	-	-	75.0%
DL#C.75	62.5	-	-	75.0%
DL#C.90	25	-	-	75.0%
DL#C.98	5	-	-	75.0%

# indicates the identification number for each subject.

L# = stands for layer in the homogenisation study.

DL# = the “D” stands for diarrhoea in the water content study; the “L” refers to samples that have been also used in the homogenisation study, that contained PBS and underwent a bead-beating step.

DL#B = samples that did not contain PBS but underwent a bead-beating step.

DL#P = samples that contained PBS but did not undergo a bead-beating step.

DL#C = samples that did not contain PBS and did not undergo a bead-beating step.

### Effect of stool homogenisation during collection

**Figure 1:**
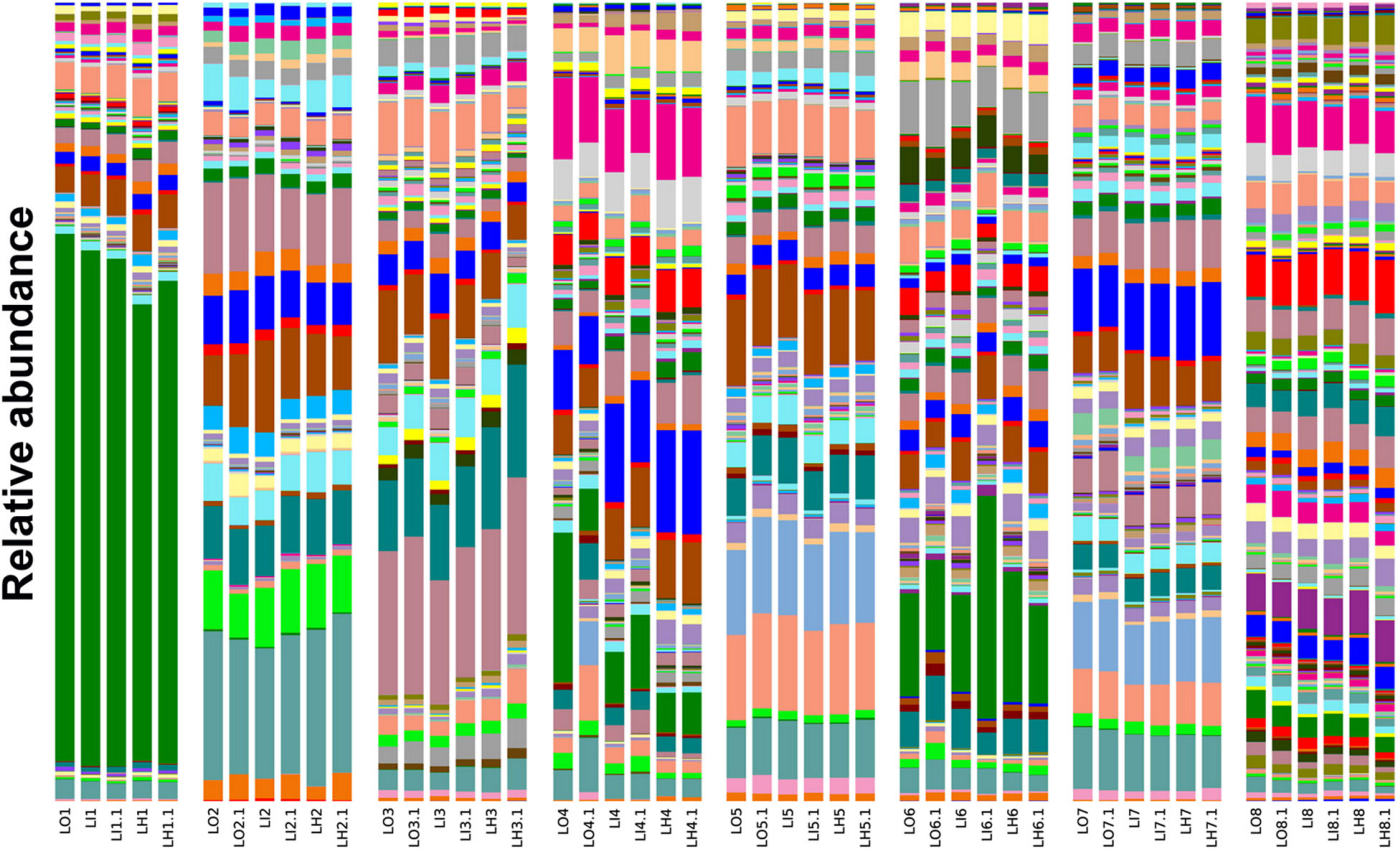
**Spatial organization of the microbial community (species level) in stool specimens.** 250 mg of stool (N = 8) was collected in the outer (LO) and inner area (LI) layer and once the stool had been homogenised (LH). Stools were collected in duplicates for each condition.

**Figure 2:**
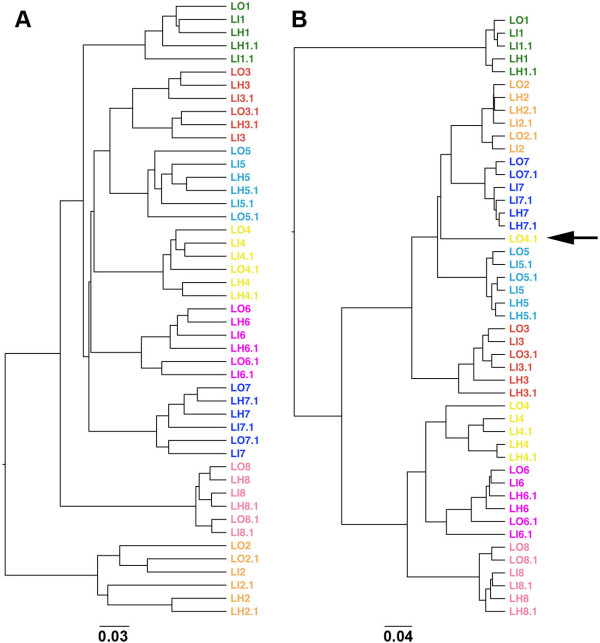
**UPGMA clustering based on weighted (A) and unweighted UniFrac (B) distance analysis.** 250 mg of stool (N = 8) was collected from the outer (LO) and inner (LI) layers and after the stool had been homogenised (LH). Stools were collected in duplicates for each condition (48 samples in total). Unweighted UniFrac allows clustering by taking into account only the microbial composition, while weighted UniFrac considers both composition and abundance of OTUs.

Usually, participants are instructed to homogenise their stool samples during collection. However, given the laborious and unpleasant nature of this task, it is possible that they might not have fully complied with this procedure. To evaluate the impact of homogenisation on the composition of the microbial community, we analysed the 48 samples as specified in the experimental design cited above (L#) by means of pyrosequencing the 16S rRNA gene at a normalised depth of 6173 sequences of 290 bp per sample. The microbial profile at the species level was quite similar between a portion of the stool collected in the outer area, in the inner area and after homogenisation, except for sample LO4.1, which showed a similar diversity but distinct abundance of Operational Taxonomic Units (OTUs) (Figure 1, Additional file 1: Table S1). This observation was confirmed by an Unweighted Pair Group Method with Arithmetic Mean (UPGMA) clustering analysis based on unweighted and weighted UniFrac distances (Figure 2). Sample LO4.1 from subject #4 was the only one that clustered far from the other samples from the same stool when both microbial composition and abundance were considered (weighted UniFrac analysis, Figure 2B).

### Effect of stool water content

To evaluate how stool water content affects the microbial community, we analysed the 46 samples from four out of the eight participants, as described in the experimental design section above.

**Figure 3:**
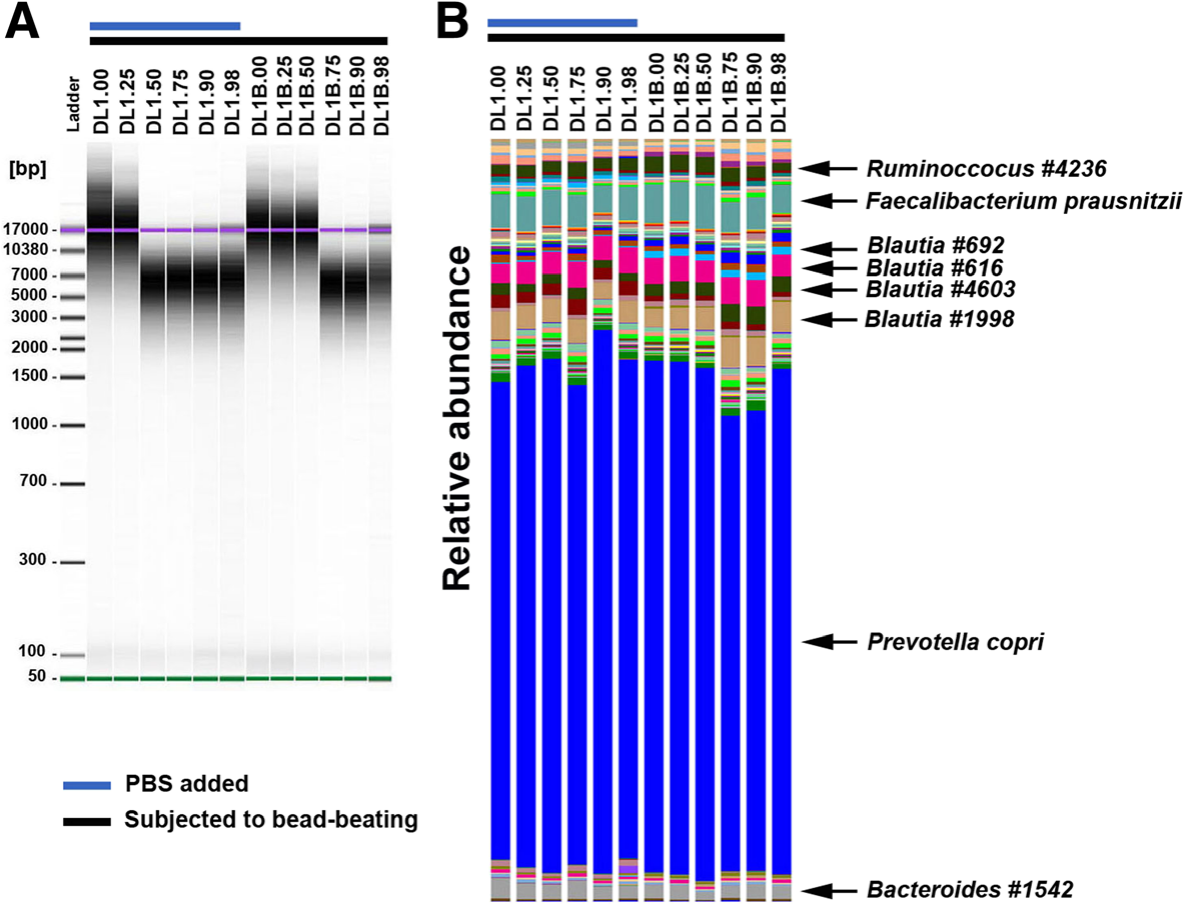
**Effect of water content on genomic DNA integrity. (A)** Gel electrophoresis analysis. For each sample, genomic DNA equivalent to 1 mg of faecal sample was loaded on an Agilent 2100 Bioanalyzer chip using the Agilent 12000 kit. DL1 corresponds to participant L1 from the homogenisation evaluation. **(B)** Microbial diversity at the species level. The taxonomic analysis was performed using a cut-off of 97% similarity. The “#” followed by a number indicates an arbitrary identifier for an unknown OTUs.

After the extraction procedure, genomic DNA was loaded in an Agilent 2100 Bioanalyzer chip in order to evaluate integrity. A comparison of the DNA extracted from DL1 samples (presence of beads and PBS) with those of DL1B’s (presence of beads but not PBS) showed that the addition of PBS caused greater genomic DNA degradation (Figure 3A). This finding was confirmed by a decrease in DNA size to lower than 10 Mbp with 125 mg of stool (sample DL1.50, Table 1) and 50% PBS. In contrast, in the absence of PBS this degradation was also observed but only when the stool weighed 62.5 mg (DL1B.75). Interestingly, we observed a double effect of stool water content and bead-beating when dealing with a small amount of stool matter.

Although the presence of PBS could increase the degradation of genomic DNA, the microbial community profile was not affected at the species level (Figure 3B). This observation could be explained by the fact that the microbial analysis was based on the PCR amplification of the V4 region of the 16S rRNA gene, which is around 300 bp, whereas the degraded genomic DNA fragments were larger than 3000 bp. Moreover, this size may be sufficient for shotgun sequencing as DNA would be cut into fragments of between 400 and 800 bp. However, further sequencing experiments are required to confirm that the gene content analysis is not biased.

### Effect of bead-beating during DNA extraction

**Figure 4:**
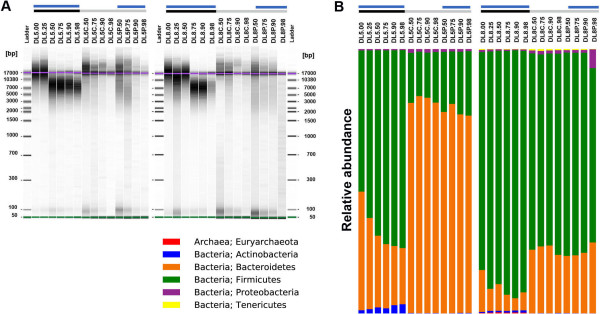
**Effect of bead-beating on genomic DNA integrity and on microbial community composition. (A)** Gel electrophoresis analysis. For each sample, genomic DNA equivalent to 1 mg of faecal sample was loaded on an Agilent 2100 Bioanalyzer chip using the Agilent 12000 kit. **(B)** Microbial diversity profile at the phylum level. Sample identification is identical to that indicated in the legend of Figure 3. DL5 and DL8 correspond to the participants L5 and L8 from the homogenisation evaluation. Samples with the identification starting with DL5C and DL8C were not subjected to bead-beating nor did they contain PBS. DL5P and DL8P contained only PBS. Black bars indicate the samples subjected to bead-beating and grey bars those that were not, while blue bars show the samples to which PBS was added.

**Figure 5:**
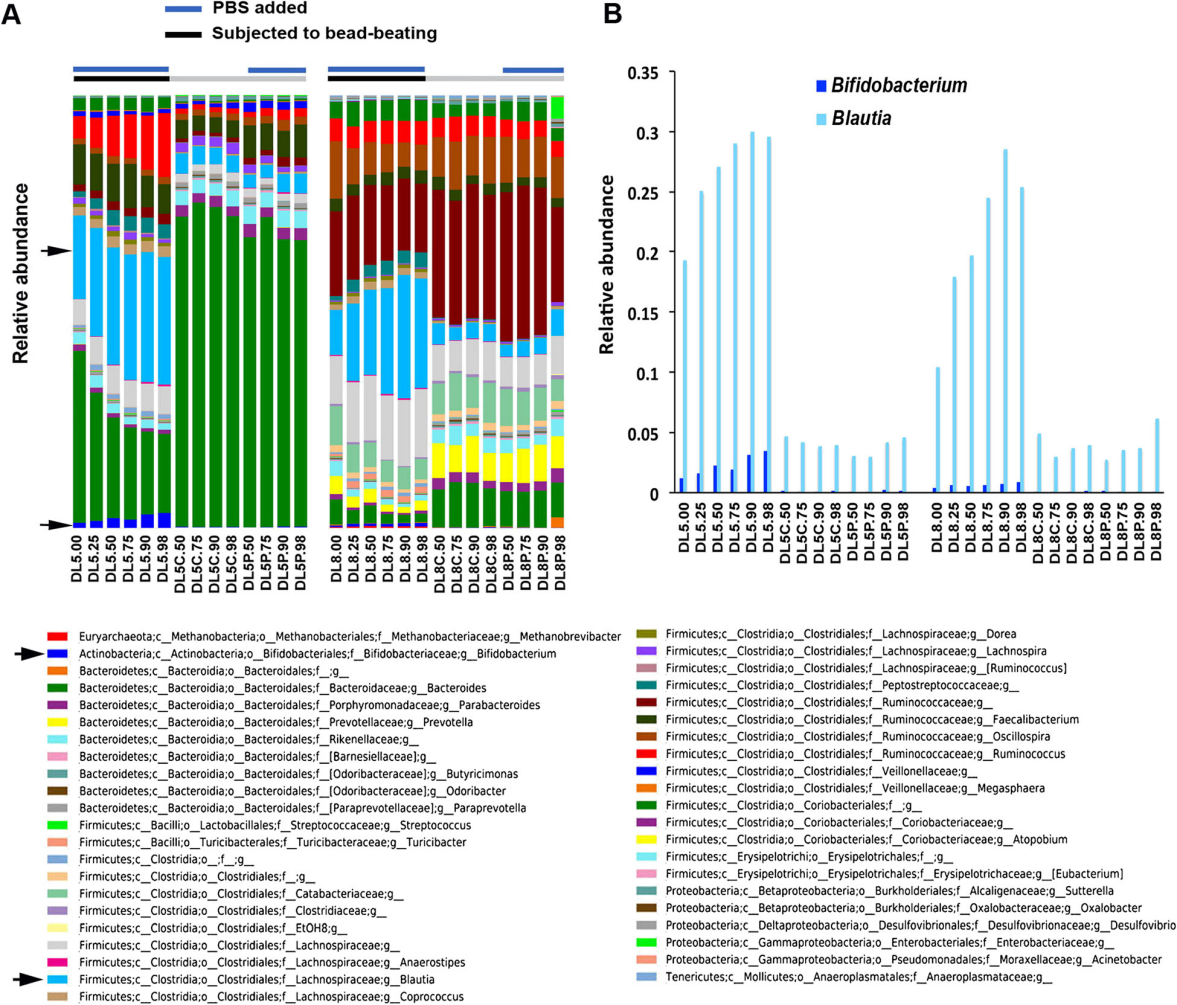
**Microbial profile at the genus level. (A)**. All OTUs are shown. The arrows indicate the detection of *Bifidobacterium* and increase in the detection of *Blautia* with the bead-beating procedure. Black bars represent the samples subjected to bead-beating and grey bars those that were not, while the blue bars indicate the samples to which PBS was added. **(B)**. Relative abundance of *Blautia* and *Bifidobacterium*. The identification of the samples is identical to that shown in the legend of Figure 3. DL5 and DL8 correspond to participants L5 and L8 from the homogenisation evaluation. Samples DL5C and DL8C represent those that were not submitted to bead-beating nor did they contain PBS. DL5P and DL8P contained only PBS.

A bead-beating step during DNA extraction is required to break down the cell wall of Gram-positive bacteria [13]. To evaluate the effect of bead-beating on the microbial community of diarrhoeic samples, we compared conditions with and without a bead-beating step, and with and without an increasing volume of PBS (samples DL5 and DL8 versus DL5P and DL8P). Although the disruption step caused degradation of genomic DNA, in an increased volume of PBS, it did not greatly modify the microbial community profile (Figure 4B). Moreover, samples containing a different volume of PBS (see samples DL5.00 to DL5.98 and DL8.00 to DL8.98) clustered together (Figure 5A and B), as shown by an UPGMA-UniFrac analysis, and presented a similar alpha diversity, as measured by phylogenetic diversity (PD) metric (Additional file 2: Figure S1). However, in the absence of bead-beating during the extraction procedure, genomic DNA did not show any sign of degradation at any volume of PBS tested, but the DNA yields were lower than with bead-beating (the average sum was 816 ng/μl versus 941 ng/μl with bead-beating). The microbial profile of these samples also differed completely to that of those subjected to bead-beating (DL# versus DL#P and DL#C; where # = 5 or 8). As expected, the absence of bead-beating significantly decreased the detection of Gram-positive bacteria such as Firmicutes and Actinobacteria phyla (Figure 4B). At the genus level, proportions of *Blautia* and *Bifidobacterium* were decreased by at least 5- and 14-fold, respectively (Mann Whitney test, p < 0.001) (Figure 5).

**Figure 6:**
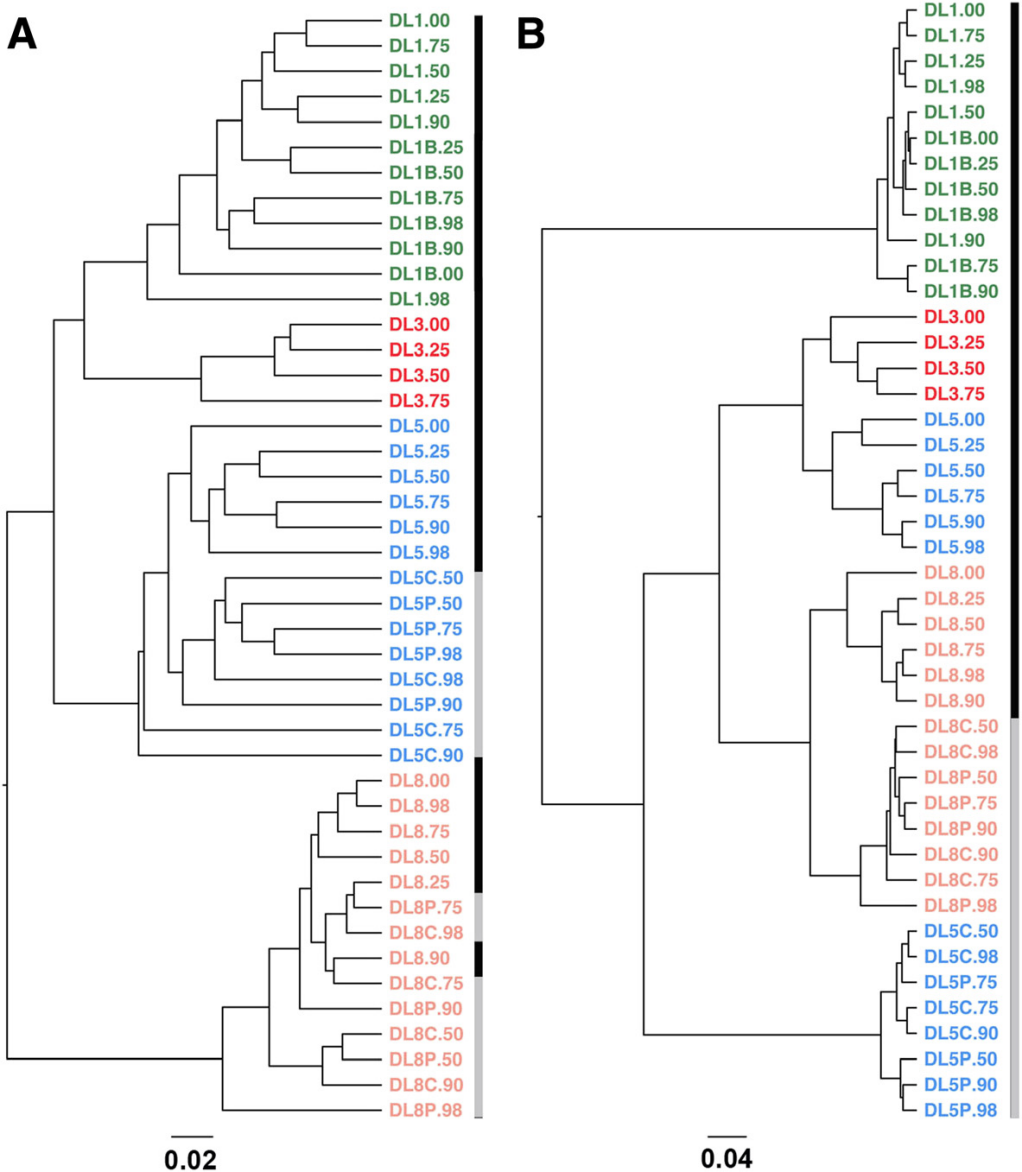
**UPGMA clustering based on weighted (A) and unweighted UniFrac (B) distance analysis.** Unweighted UniFrac allows the clustering of samples by taking into account only the microbial composition, whereas weighted UniFrac considers both composition and abundance of OTUs. Black bars also indicate the samples subjected to bead-beating and grey bars those that were not.

The UPGMA clustering analysis based on the unweighted UniFrac method, which takes into account the microbial composition, did not show separation of the samples with or without a bead-beating step (Figure 6A). However, when the analysis was based on a weighted UniFrac method, which considers both microbial composition and abundance, samples from one of the four subjects clustered separately (Figure 6B). Here we show that the inclusion of this procedure dramatically changed both the migration profile of the genomic DNA and the taxonomic profile of stool samples.

## Conclusion

Microbial community studies involve a variety of procedures, ranging from sample collection to sequence data interpretations. Given the increasing relevance of metagenomics for research into intestinal disorders, it is crucial that the data generated in each study be optimally comparable across all those already underway. However, strong biases can be introduced into stool research, in particular during stool collection and storage and during DNA extraction. We previously recommended that stool samples be kept at room temperature and be brought to the laboratory within 24 h after collection or alternatively be stored immediately at -20°C by the volunteer in a home freezer, to be later transported in a freezer-pack to the laboratory, where all samples are stored at -80°C before further treatment [14].

Our findings from the present study indicate that homogenisation of the stool during collection is recommendable but not indispensable. Indeed, samples collected from the inner and outer layers of stool samples showed a similar microbial composition and abundance. Moreover, we show that the percentage of water typically found in diarrhoeic samples does not affect the clustering of samples from the same subjects. To validate our results, analysis of diarrhoeic samples could be compared with non-diarrhoeic ones from the same individual; however, the collection of these two types of samples from the same healthy subjects would be complicated for ethical reasons. Moreover, since other next generation sequencing platforms will allow a greater sequencing depth, this may allow a deeper characterization of the microbial community and could reveal additional differences in the microbial community composition for the various conditions measured in this study.

Finally, our study also reveals that microbial disruption by bead-beating allows greater detection of Gram-positive bacteria such as *Blautia* (Firmicutes phylum) and *Bifidobacterium* (Actinobacteria phylum), commonly detected in human stools. In conclusion, the hydration of faecal samples and their degree of homogenisation do not significantly alter their microbial community composition and structure. However, although the mechanical disruption of microbial cells causes genomic DNA degradation in simulated diarrhoeic stool samples, our findings confirm that this step is necessary for the detection of Gram-positive bacteria such as *Blautia* and *Bifidobacterium*.

## Methods

### Ethics statement

Subjects provided their written consent to participate in this study, and the Institutional Review Board of the Vall d’Hebron Hospital (Barcelona, Spain) approved this consent procedure.

### Sample collection protocol

Stools were collected from eight healthy participants. The collection protocol involved providing participants with an ice bag containing an emesis basin (Ref. 104AA200, PRIM S.A, Spain), a 50-mL sterile sampling bottle (Ref. 409526.1, Deltalab, Spain), a sterile spatula (Ref. 441142.2, Deltalab, Spain), and gloves (Additional file 3: Figure S2) during their visit to the laboratory. For the purpose of stool collection, the participants were instructed to do the following once at home: 1) use the emesis basin to collect the stool; 2) after the deposit, transfer it to the sampling bottle ensuring no homogenisation; 3) take it to the lab within the first 3 hours after deposit; and 4) in the laboratory, the samples were processed as mentioned in the experimental design, and then the samples were stored at -80°C.

### Naming convention

Since the samples from same individuals were used to test different factors that could affect microbial composition, a labeling nomenclature had to be settled down as indicated in Table 1. The “D” stands for “diarrhoea” in the water content study. The “L” stands for “layer”, “O” for “outer" and “I” for the “inner" layer of the stool, and “H” for “homogenised stool” in the homogenisation evaluation. The “P” stands for samples that contained PBS to simulate diarrhoea not undergoing bead-beating, while “B” stands for samples that did not contain PBS, but underwent bead-beating. Samples with the “C” label are controls that did not contain PBS and did not undergo bead-beating. The numbers 1–8 signify the 8 different volunteers.

### Genomic DNA extraction

To evaluate the need for stool homogenisation during collection, aliquots (250 mg) of each sample were suspended in 0.1 M Tris (pH 7.5), 250 μl of 4 M guanidine thiocyanate, 40 μl of 10% N-lauroyl sarcosine and 500 μl of 5% N-lauroyl sarcosine, as previously described in [15]. DNA extraction was carried out by mechanical disruption of the microbial cell wall using beads (Lysing matrix E, MP Biomedicals, Spain). The disruption was performed by shaking the mixture using the Bead-Beater-8 (BioSpec, USA) at a medium speed of about 1500 oscillations/min for 3 minutes, followed by 3 minutes in ice and again followed by 5 minutes at a medium speed of about 1500 oscillations/min. Finally, nucleic acids were recovered from clear lysates by alcohol precipitation.

To evaluate the effect of stool water content and a bead-beating step, aliquots of samples were homogenised with various volumes of PBS (final weight of 250 mg) and with or without beads, as described in Table 1. They were then processed the same way as described above. In samples in which beads were not used, the bead-beater step was also omitted.

After genomic DNA extraction, an equivalent of 1 mg of each sample was used for DNA quantification using a NanoDrop ND-1000 Spectrophotometer (Nucliber). DNA integrity was examined by microcapillary electrophoresis using an Agilent 2100 Bioanalyzer with the DNA 12000 kit, which resolves the distribution of double-stranded DNA fragments up to 17,000 bp in length.

### Microbial community analyses

#### 454 pyrosequencing of the V4 variable region of the 16 S rRNA gene

To analyse bacterial composition, we subjected extracted genomic DNA to PCR-amplification of the V4 hyper-variable region of the 16S rRNA gene. On the basis of our analysis done using PrimerProspector software [16], the V4 primer pairs used in this study were expected to amplify almost 100% of the Archaea and Bacteria domains. The 5’ ends of the forward primer V4F_517_17 (5′-GCCAGCAGCCGCGGTAA-3′) [17] and the reverse primer V4R_805_19 (5′-GACTACCAGGGTATCTAAT-3′) [18] were tagged with specific sequences for pyrosequencing as follows: 5′-CCATCTCATCCCTGCGTGTCTCCGACTCAG-{MID}-{GCCAGCAGCCGCGGTAA}-3′ and 5′ CCTATCCCCTGTGTGCCTTGGCAGTCTCAG-{GACTACCAGGGTATCTAAT}-3′. Tag pyrosequencing was performed using multiplex identifiers (MIDs) (Roche Diagnostics) of 10 bases, which were specified upstream of the forward primer sequence (V4F_517_17). Standard PCR amplification was run in a Mastercycler gradient (Eppendorf) at 94°C for 2 min, followed by 35 cycles of 94°C for 30 sec, 56°C for 20 sec, 72°C for 40 sec, and a final cycle of 72°C for 7 min. PCR products were purified using a PCR Purification kit (Qiagen, Spain) and subsequently sequenced on a 454 Life Sciences (Roche) FLX system (Scientific and Technical Support Unit, Vall d’Hebron Research Institute, Barcelona, Spain), following standard 454 platform protocols.

#### 16S rRNA sequence data analysis

A total of 1.47 million sequence reads from 96 samples were analysed using the default settings in the Quantitative Insights Into Microbial Ecology (QIIME) package of software tools [19]. The 16S rRNA sequences were quality-filtered and demultiplexed. These reads had an average length of 290 bp. Using the pick-otus protocol, we classified the sequence reads into OTUs on the basis of sequence similarity. Sequence reads were then clustered against the February 2011 release of the Greengenes 97% reference dataset (http://greengenes.secondgenome.com) [20,21]. Taxonomy was assigned using the Basic Local Alignment Search Tool (BLAST) [22]. The representative sequences of all OTUs were then aligned to the Greengenes reference alignment using PyNAST [18], and this alignment was used to construct a phylogenetic tree using FastTree [23] within QIIME. The resulting tree topology with associated branch lengths was used for subsequent diversity analyses (for many downstream analyses, samples were rarefied at 6173 and 9390 sequences per sample for the homogenisation and for the water content evaluations, respectively). One sample (LO1.1) was removed from the analysis because of low count reads. Alpha diversity was estimated using the phylogenetic diversity metric. Beta diversity analysis was performed using the UPGMA clustering method based on weighted and unweighted UniFrac distances [24].

### Availability of supporting data

Sequences have been deposited in NCBI database with the accession number SRP040438.

## Abbreviations

PBS: Phosphate-buffered saline; UPGMA: Unweighted pair group method with arithmetic mean; QIIME: Quantitative insights into microbial ecology; OTU: Operational taxonomic units; BLAST: Basic local alignment search tool.

## Competing interests

The authors declared that they have no competing interests.

## Authors’ contributions

CM, AS and SP conceived and designed the study and drafted the manuscript. AS, SP and GM carried out the experiments. CM and XM did the 16S data generation and analysis. FA, JD and FG participated in design and coordination of the project. All authors read and approved the final manuscript.

## Supplementary Material

Additional file 1: Table S1 Legend of Figure 1. Click here for file

Additional file 2: Figure S1 Alpha-diversity curves at a number of rarefaction depths. Each line represents the results of the alpha-diversity phylogenetic diversity whole tree metric (PD whole tree in QIIME) for all samples from subjects #5 and #8. Click here for file

Additional file 3: Figure S2 Kit for stool collection (see the method section). Click here for file
