# Supplementary figures and images for: Processing faecal samples: a step forward for standards in microbial community analysis
Source: BMC Microbiol. 2014 May 1;14:112. doi: 10.1186/1471-2180-14-112 (PMC4021188; doi:10.1186/1471-2180-14-112)

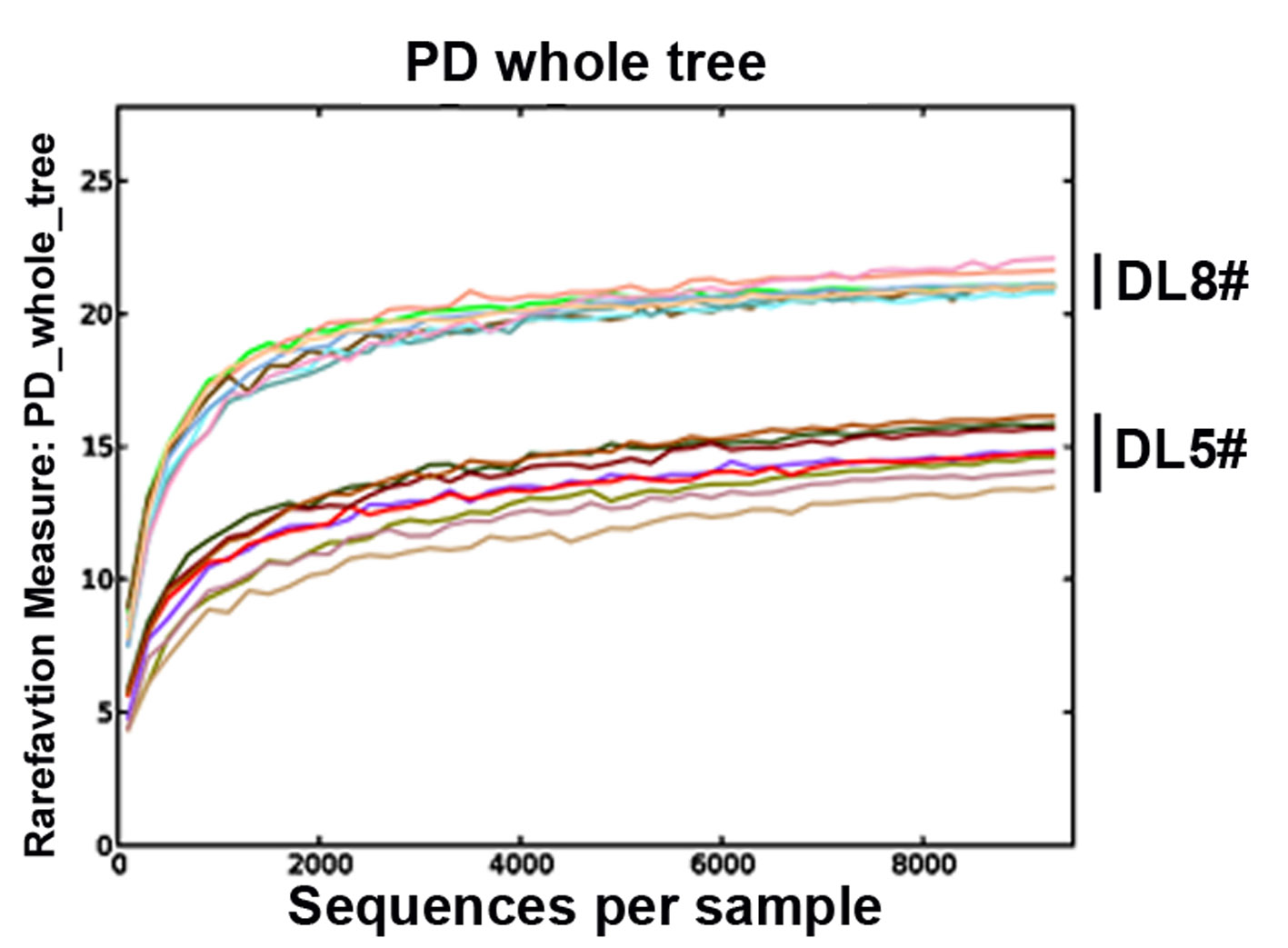

Supplement: Additional file 2: Figure S1 — Alpha-diversity curves at a number of rarefaction depths. Each line represents the results of the alpha-diversity phylogenetic diversity whole tree metric (PD whole tree in QIIME) for all samples from subjects #5 and #8. [file 1471-2180-14-112-S2.png]

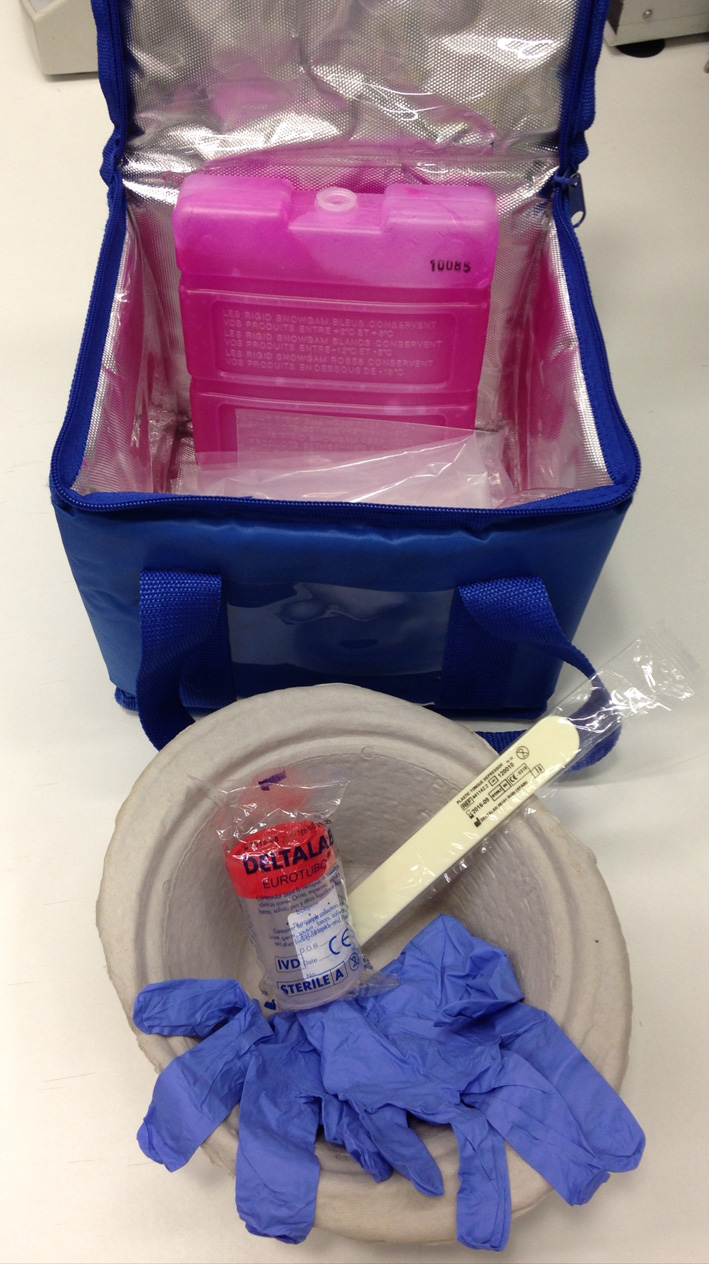

Supplement: Additional file 3: Figure S2 — Kit for stool collection (see the method section). [file 1471-2180-14-112-S3.png]
